# Fidelity of Implementation of an Evidence-Based HIV Prevention Program among Bahamian Sixth Grade Students

**DOI:** 10.1007/s11121-014-0486-y

**Published:** 2014-04-16

**Authors:** Bo Wang, Lynette Deveaux, Valerie Knowles, Veronica Koci, Glenda Rolle, Sonja Lunn, Xiaoming Li, Bonita Stanton

**Affiliations:** 1 Pediatric Prevention Research Center, Department of Pediatrics, Wayne State University School of Medicine, 4707 St. Antoine, Suite W534, Detroit, MI 48201 USA; 2 Office of HIV/AIDS, Ministry of Health, Shirley Street, Nassau, The Bahamas; 3 Ministry of Education, Thompson Boulevard, PO Box N-3913, Nassau, The Bahamas

**Keywords:** Implementation research, HIV prevention, Fidelity, Adolescents, Bahamas

## Abstract

The Bahamian Ministry of Education has elected to implement at a national level in all Bahamian government grade six classes an evidence-based HIV prevention intervention [Focus on Youth in the Caribbean (FOYC)]. This study explores fidelity of implementation of the intervention, factors that may influence implementation fidelity, and the impact of variations in the implementation fidelity on student outcomes. Data were collected in the first wave of national implementation in 2011, involving 35 government primary schools and 110 teachers and 2,811 students. Structural equation modeling was performed to examine the relationships among factors which facilitated or impeded teachers’ implementation of FOYC. Results indicate that teachers taught 16.3 out of 30 core activities, 24.9 out of 46 total activities, and 4.4 out of 8 sessions on average. The strongest predictor of implementation fidelity was teacher comfort level with the FOYC curriculum. Teachers who did not perceive the FOYC intervention to be important for their students or who had attended only part of a FOYC training workshop were more likely to change the curriculum. Increased duration of experience as a teacher (>10 years) was negatively associated with fidelity of implementation. Teacher’s perception of the importance of the FOYC intervention and implementation fidelity had direct positive effects on students’ HIV/AIDS knowledge, reproductive health skills, protective intentions, and self-efficacy. Youth did not appear to benefit from FOYC if two or fewer sessions were delivered. We concluded that an evidence-based HIV prevention intervention can be implemented at a national level. Prior training of teachers in the intervention curriculum, teacher perception of the importance of the intervention, and fewer years as a teacher are associated with implementation fidelity. Implementation fidelity is associated with improved student outcomes.

## Introduction

Significant progress has been made in the field of HIV prevention over the past quarter-century. Forty-four “best evidence” effective HIV prevention programs have been identified by the Centers for Disease Control and Prevention’s “Prevention Synthesis Project” (Centers for Disease Control and Prevention CDC 2011). Among these best-evidence interventions, seven target high-risk adolescents including four small-group risk reduction interventions (e.g., Sisters Saving Sisters), two clinic-based intervention (e.g., HORIZONS), and one community-based intervention [Focus on Youth (FOY) + Informed Parents and Children Together (ImPACT)]. As important as this research is, evidence-based interventions make little contribution to HIV prevention efforts until they are successfully put into practice (Rebchook et al. 2006). The Centers for Disease Control and Prevention (CDC) has made great efforts in transferring effective HIV prevention research to community prevention practice by identifying, packaging, and disseminating effective behavioral interventions (Collins et al. 2006; Lyles et al. 2007), yielding implementation of an increasing number of evidence-based interventions among expanded audiences.

While increased implementation of evidence-based behavioral interventions is critical to public health outcomes, this important step of translational research faces new challenges. Evidence-based behavioral interventions were developed and evaluated in specific settings with unique characteristics under controlled conditions; their continued effectiveness under varied, complex, and possibly less predictable conditions cannot be presumed (Collins et al. 2006). Imbedded in this recognition are the two questions. First, what aspects of the intervention are necessary for its continued success regardless of where it is delivered? These critical aspects of the intervention are commonly referred to as “core elements” (Galbraith et al. 2009; Kelly et al. 2000). Second, what aspects of the intervention can be (or perhaps even should be) altered, deleted, or substituted in differing settings? These two activities—one preserving the intervention as it was originally delivered and the other adapting it to new settings—are both hypothesized to be critical to implementation efforts, but may also be at odds with each other. Determining the importance of so-called “fidelity of implementation” requires more definition around the intervention that is being delivered, including what can and what cannot be altered. A definition of “fidelity of implementation” increasingly being utilized refers to the degree to which program providers implement programs as intended by the program developer, e.g., the “core elements” as viewed by the developer (Dearing 2009; Dusenbury et al. 2003). While other definitions have been offered, this definition has the advantages of being definable and able to serve as a platform for testable hypotheses. As an example of use of the core elements to evaluate fidelity, the Mpowerment Project (MP), an evidence-based intervention for young men having sex with men, was implemented by 69 Community-based organizations (CBOs) in 45 states throughout the USA (Rebchook et al. 2006). The program developers identified nine core elements, and implementation evaluation found that each of the core elements was dropped by 20 to 45 % of CBOs (e.g., 27 % dropped “program publicity”) and modified by 30 to 50 % of the CBOs (e.g., 50 % modified “venue-based outreach”) (Rebchook et al. 2006). Likewise, Focus on Kids (FOK), an efficacious HIV prevention program for high-risk adolescents, had been disseminated both nationally in the USA and internationally (including countries in Asia, Africa, and Latin America). Program developers identified eight core elements; a subsequent telephone survey among community providers revealed that on average only half of the core elements (4.3 out of 8 core elements) were being implemented (Galbraith et al. 2009).

Based on the belief that fidelity of implementation (however defined) is important to outcomes, implementation scientists have identified a range of factors which appear to increase or decrease implementation fidelity: teacher training, program characteristics, teacher characteristics, and organizational characteristics (Dusenbury et al. 2003). Teacher (and program provider) training has long been recognized as an essential element of successful implementation of prevention curricula (Payton et al. 2000; Perry et al. 1997). A study of Teenage Health Teaching Modules found that teachers who received training taught the curriculum with a greater fidelity compared with teachers who did not receive training (Parcel et al. 1991). However, some studies have suggested that only extensive training is associated with higher fidelity of implementation (Dusenbury et al. 2003; Perry et al. 1990). Little is known about how training actually impacts student outcome. A number of teacher characteristics have been found to be related to fidelity of implementation including teacher’s positive attitude toward prevention programs (Beets et al. 2008), shorter duration of time as teacher (Rohrbach et al. 1993), and confidence in their ability to teach interactive methods (Rohrbach et al. 1993). Organizational characteristics including receptivity to the prevention program, support by the principal or other school administrators, and the organization’s readiness to implement new programs have been associated with increased fidelity of implementation (Beets et al. 2008; Dusenbury et al. 2003). Program provider’s perception of community *ownership* of the intervention (e.g., a belief that the intervention addresses a local issue and that they or their community had significant input into the development of the intervention) is positively associated with the fidelity of program implementation (Draper et al. 2010). A perception by the teacher that the intervention is at least as important for the students as potentially competing alternatives (such as reading) (Rogers 2002) and students’ apparent engagement in the intervention curriculum appears to promote implementation fidelity (Mihalic et al. 2008).

Although overall HIV prevalence has been declining (from 4.1 % in 1999 to 2.8 % in 2011) in The Bahamas in the past decade, the rate of 1.2 % among youth ages 15 to 24 years remains concerningly high (International Group 2010; UNAIDS 2012). Heterosexual activity is the predominant mode of transmission. Focus on Youth (FOY) with Informed Parents and Children Together (ImPACT) was selected by CDC’s Prevention Research Synthesis project as a Best Evidence Program and is included in the CDC’s “Diffusion of Behavioral Interventions” portfolio (Lyles et al. 2007). Project developers, in collaboration with the CDC’s DEBI staff, identified the “core elements” of FOY and ImPACT (Galbraith et al. 2009). Over the last decade, the US-Bahamian team developed and evaluated the Bahamian adaptation of FOY and ImPACT [a ten-session plus two booster sessions-adolescent HIV prevention intervention entitled “Focus on Youth in the Caribbean” (FOYC) and a 1-hour parental monitoring intervention entitled “Caribbean Informed Parents and Children Together” (CImPACT)] (Deveaux et al. 2007). The core elements of FOY/FOYC are depicted in Table 1. A randomized, controlled, intervention trial was conducted among 15 elementary schools in The Bahamas. Short- and long-term evaluations showed that the intervention significantly increased youth’s HIV/AIDS knowledge, condom use skills, perceptions, intentions, and practices among Bahamian preadolescents (Gong et al. 2009; Stanton et al. 2012). Based on the effectiveness of the intervention through 36 months, the Bahamian Ministry of Education (MOE) decided to implement FOYC + CImPACT in all grade six classes in the government elementary schools throughout the nation, with follow-up booster sessions to be delivered in grades seven and eight in the government junior high schools. FOYC is delivered as part of the Health and Family Life Education (HFLE) curriculum, and CImPACT is incorporated into parent-teacher meetings (Knowles et al. 2012). The MOE decided to reduce the number of sessions from ten to eight to conserve curricular time, but worked with the FOYC developers to ensure that no core elements were dropped.

**Table 1:** Core elements of Focus on Youth (FOY) and Focus on Youth in the Caribbean (FOYC)

Core elements	
1	Delivering intervention to youth in community-based settings.
2	Using skilled facilitators to implement the youth group session.
3	Use existing groups (such as a “friendship groups” or classroom) to strengthen peer support.
4	Using culturally appropriate interactive activities proven as effective learning strategies to help youth capture the important constructs in the theory.
5	Including a “family tree” to contextualize and personalize abstract concepts such as decision-making and risk assessment.
6	Enabling participants to learn and practice a decision-making model.
7	Training participants in assertive communication and refusal skills specifically related to negotiation of abstinence or safer sex behaviors.
8	Teaching youth proper condom use skills.

Implementation of evidence-based HIV prevention programs (and analyses thereof) at a national level remains uncommon worldwide (Brown et al. 2013). National implementation of FOYC in The Bahamas offers a unique opportunity to explore important issues arising in the implementation of an effective HIV intervention. Drawing on data gathered through the first wave of national implementation, this analysis addresses three research questions: (1) To what extent did the teachers implement the FOYC intervention with fidelity in their classes?; (2) What factors influenced the teacher’s implementation fidelity and what is the relationship between influencing factors?; and (3) How does implementation fidelity impact students’ outcomes?

## Method

### Study Site

In the fall of 2011, all 35 elementary schools located on three of the Bahamian islands participated in the first wave of national implementation. Twenty-three of the schools were located in Island #1 (I-1), nine schools in Island #2 (I-2), and three schools in Island #3 (I-3). The 35 participating schools housed 110 grade six classes and teachers: I-1 housed 88 (80 % of the total) teachers; I-2 housed 18 (16.4 %) teachers; and I-3, housed 4 (3.6 %) teachers. The present analysis is based on Wave 1 data that were collected from the three most populated islands. The research protocol was approved by the Wayne State University Human Investigation Committee and the Institutional Review Board of the Bahamian Princess Margaret Hospital, Public Hospitals Authority.

### Teacher Training

The MOE established three teacher training workshops following the protocol used by the DEBI program to train future interventionists in the delivery of FOY in two major participating islands (i.e., I-1 and I-2). The teacher training covered the following: (1) review of the need for HIV prevention in The Bahamas; (2) overview of FOYC including the research showing its effectiveness; (3) a walk-through of each of the eight sessions of FOYC with participation and “role-play” of the core activities considered to be critical to the success of FOYC; and (4) a didactic question-and-answer period regarding contraception and condom use. All 110 teachers (regardless of attendance at a workshop) were given a copy of the FOYC teacher training manual.

A total of 65 (59.1 %) teachers attended a FOYC training workshop in 2011. Twenty-seven (24.5 %) teachers reported that they had attended a prior FOYC workshop before 2010 when they participated in the original FOYC intervention study. Overall, 77 (70 %) teachers received training supporting their delivery of the FOYC curriculum; 33 teachers (30 %) received no training.

### Measures

#### Teacher’s Fidelity of Implementation

To assess fidelity of implementation, all teachers were asked to complete a Teacher Implementation Checklist specific for each of the eight sessions of FOYC after they had taught the session. The checklist includes the 30 activities identified by the developers as “core elements” and an additional 16 activities. The teachers documented the activities that they covered in each session, and for the ones taught, their degree of comfort in teaching the lesson (very comfortable, rather comfortable or not comfortable), whether they had modified the format of the activity outlined in the manual, and how many students (most, some, and few) appeared to be engaged in the lesson. A mean score was calculated for teacher’s level of comfort in teaching the lesson, frequency of modifying the FOYC activities, and student engagement across each core activity. Fidelity of implementation is defined as adherence to the core activities in this analysis. Because it is possible that the other activities that are not currently identified as core activities may impact student outcomes, adherence to all activities in the curriculum was also assessed as was the number of sessions taught.

#### Teacher’s Characteristics, Training Experience, and Perceptions

A brief pre-implementation questionnaire was used to collect information described in the extant research summarized in the Introduction section as influencing fidelity of intervention implementation: teacher’s level of formal education; years as a teacher/guidance counselor; teacher’s attendance at FOYC training workshop; teachers’ perceptions of the importance of HIV prevention (very important and somewhat important or not important) for grade six students in their community or schools; teacher’s confidence in teaching the FOYC intervention; and teacher’s sense of “ownership” of the curriculum (i.e., perceiving it as a “Bahamian intervention”) (Beets et al. 2008; Draper et al. 2010; Dusenbury et al. 2003; Rohrbach et al. 1993). In bivariate analyses, responses were grouped into two collapsed categories for years as a teacher/guidance counselor (1–10 years and >10 years), number of days of training attended (4–5 days and 3 days or less), and perceptions of the importance of HIV prevention (very important and somewhat/not important) due to low frequencies in some categories.

#### Student Outcomes

An anonymous curricular assessment instrument, adapted by the MOE from a version of the Bahamian Youth Health Risk Behavioral Inventory (BYHRBI) (Deveaux et al. 2011), was administered to grade six students at the beginning of grade six before receipt of FOYC and at the end of grade six. The instrument assessed HIV/AIDS knowledge and preventive reproductive health skills, as well as some perceptions, intentions, and self-reported behaviors. A 15-item scale including true and false statements was used to assess level of *HIV/AIDS knowledge* (e.g., “A person will not get HIV if he or she is taking antibiotics”). The internal consistency of the scale was high (Cronbach’s α = 0.84). Correct responses were scored 1 and incorrect 0, resulting in a summary score of 0 to 15 for each participant. *Preventive reproductive health skills* were assessed through an adaptation of the Condom-use Skills Checklist (e.g., “Check the expiration date on the back of the condom packet before using it”) (Stanton et al. 2009). The validated scale includes true and false statements describing the steps of correct condom use from opening a condom pack for use to disposal after use. This six-item scale demonstrated adequate internal consistency (Cronbach’s α = 0.83). Correct responses were scored 1 and incorrect 0, resulting in a summary score of 0 to 6 for each participant. A three-item scale was used to assess *self-efficacy* for using pregnancy/STI prevention methods (e.g., “I could get condoms”). All three items employed a yes/no response scale, with 1 point assigned for each “yes” response. Individual item scores were added to yield a summary score (range 0 to 3). The internal consistency of the scale was 0.71. A composite score was calculated as a mean score across the three items (range 1 to 3). *Intention to use condom protection* was measured using the question, “if you were to have sex in the next six months, how likely is it that you or your partner would use a condom?” Youth rated the likelihood on a 5-point Likert scale ranging from 1 (very unlikely) through 5 (very likely).

### Analysis

Frequency distribution of numbers of sessions taught, number of core activities completed, and number of all activities completed was calculated. Histograms were then constructed from frequency tables to graphically display teachers’ implementation of the FOYC intervention. To identify factors that are associated with teachers’ fidelity of implementation, we conducted a bivariate analysis (ANOVA and Student *t* test) to compare the number of core activities (from among a total of 30 possible), the number of all activities (from among a total of 46 possible), and the number of sessions taught (from among a total of eight possible) by teachers according to personal characteristics, training experience, and perceptions. These teacher-level analyses controlled for the clustering effect of school (teachers within schools).

Pearson correlation analysis was conducted to examine the associations between factors influencing teacher’s fidelity of implementation, implementation fidelity, and student outcomes. The anonymous student questionnaires were not linked at the level of the individual student; the questionnaires were however linked to the teacher (classroom). Thus, we calculated average scores of student outcomes for each classroom in correlation analysis.

Given the hierarchical nature of our data (students clustered within classes in 35 schools), mixed-effects modeling was conducted to examine the association of teacher’s fidelity of implementation with student outcomes (including HIV/AIDS knowledge, reproductive health skills, self-efficacy, and intention to use protection). Independent variables included implementation fidelity (i.e., number of core activities completed), student’s age, sex, and baseline student outcomes. School and class were included as random effect variables in the model. Regression coefficients were calculated for all variables. Bivariate analyses and mixed-effect modeling were performed using SAS 9.3 statistical software package (SAS Institute Inc., Cary, NC, USA).

Structural equation modeling (SEM) analysis was conducted to examine the relationships among factors influencing teacher’s fidelity of implementation and student outcomes using the Mplus 7 with multilevel add-on. A starting model (fidelity model) was estimated to investigate the interrelationships among factors influencing fidelity of implementation and their direct and indirect effects on fidelity of implementation. Subsequently, a full model was constructed by including student outcome latent variable into the revised fidelity model; regression paths were systematically added among the established latent variables (fidelity of implementation and student outcome) and the observed variables that influence implementation. Since students were clustered within classes in 35 schools, the cluster option in Mplus was used to correct for the potential underestimation of standard errors (Muthén and Muthén 2012). Standardized regression coefficients for all paths were estimated using robust maximum likelihood (MLR) estimation. Missing data were handled using full information maximum likelihood (FIML). The Sobel test was used to test the significance of the mediation effect. Goodness of model fit was assessed using chi-square/degrees of freedom ratio (*χ* ^2^/df), root mean square error of approximation (RMSEA), Bentler’s comparative fit index (CFI), and Tucker Lewis index (TLI) (Hatcher 2005). Acceptable model fit was determined by an RMSEA less than 0.08, values of CFI and TLI greater than 0.90, and a *χ* ^2^/df ratio less than 3 (Hatcher 2005; Hu and Bentler 1999). All presented path coefficients were standardized, and effects were considered significant at α < 0.05.

## Results

### Teacher’s Actual Implementation of FOYC Intervention

**Fig. 1:**
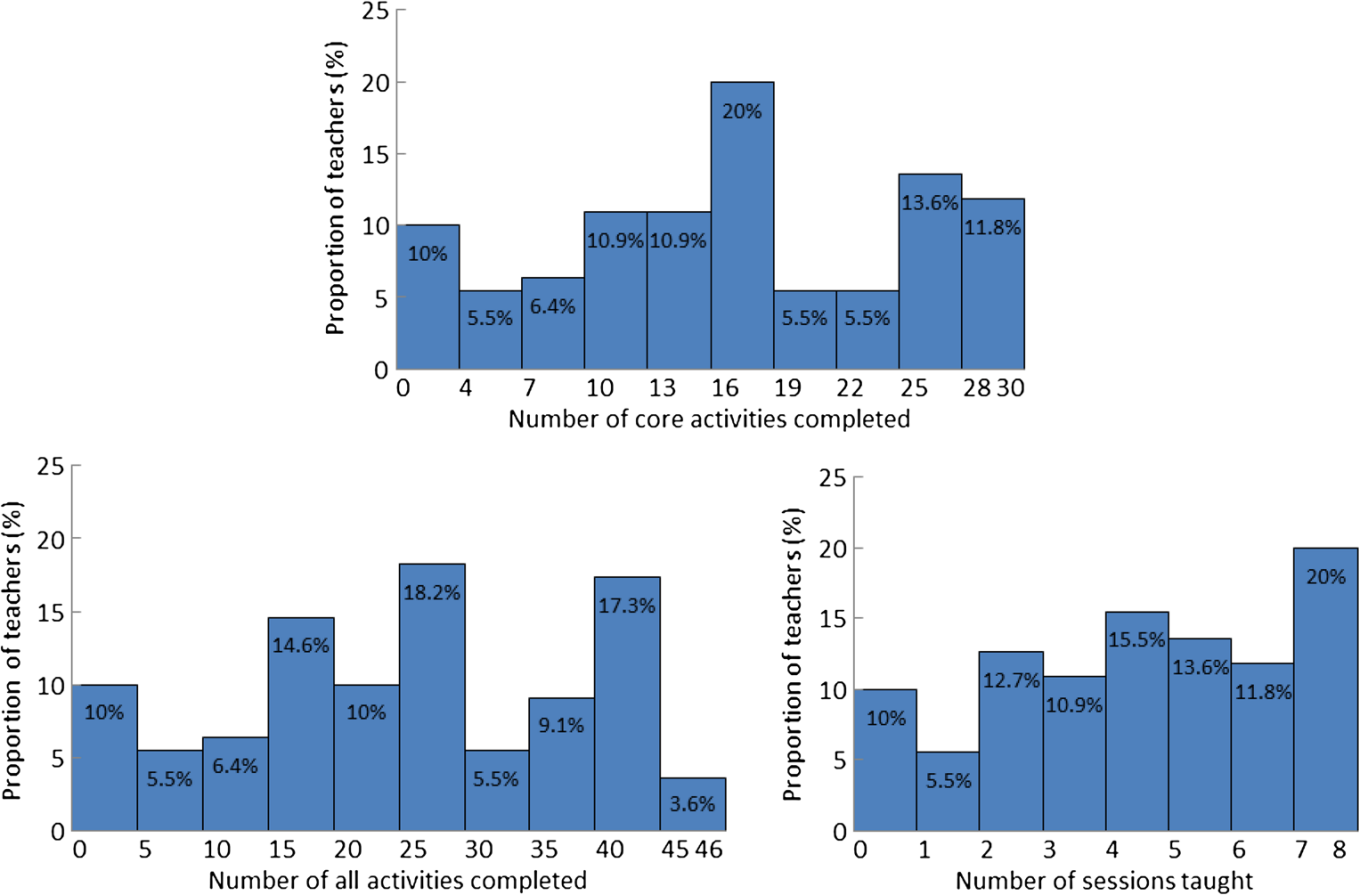
Number of core activities, all activities and sessions in FOYC curriculum taught by 110 teachers

Number of core activities, “all activities” (including core and non-core activities) and sessions completed by teachers, are displayed in Fig. 1. On average, teachers taught 16.3 core activities (SD = 8.7) from among 30 core activities, 24.9 all activities (SD = 13.6) from among 46 total activities, and 4.4 sessions (SD = 2.3) out of 8 sessions. Only 2 (1.8 %) teachers completed all core activities and covered all eight sessions, while 9 (8.2 %) teachers did not teach any activities in their classes. Thirteen (11.8 %) taught 28 or more core activities; 23 (20.9 %) taught 40 or more all activities; 22(20 %) teachers taught 7or 8sessions of FOYC curriculum. Overall, 17 (15.5 %) teachers taught less than 8 core activities (and less than 2 sessions).

Nine observers observed 138 sessions taught by the teachers during Wave 1 implementation period. These observers reported that many teachers added content to the intervention including additional reading assignments (48 sessions, 34.8 %); classroom “skits” performed by students to illustrate points such as communication (42 sessions, 30.4 %); writing assignments on a related topic (38 sessions, 27.5 %); viewing a relevant additional video (18 sessions, 13 %); identifying or creating and then singing a song (13 sessions, 9.4 %); and assigning additional homework exercises related to an FOYC topic (12 sessions, 8.7 %).

### Association between Teacher’s Characteristics, Training Experience, Perceptions, and Teacher’s Fidelity of Implementation

Table 2 presents average number of core activities, all activities and sessions taught by teachers with different personal characteristics and training experience. Teachers who had worked as an educator for more than 10 years taught fewer core and all activities and fewer sessions than teachers who only worked for less than 10 years (core activities 15.3 vs. 19.1, *t* = 2.04, *p* < 0.05; all activities 23.4 vs. 29.1, *t* = 1.98, *p* < 0.05; and sessions 4.1 vs. 5.1, *t* = 1.95, *p* = 0.05). Teachers who completed the FOYC training workshop taught more core and all activities than did the teachers who did not attend or only attended part of a training workshop (core activities 18.5 vs. 14.7, *t* = 2.01, *p* < 0.05; all activities 28.5 vs. 22.4, *t* = 2.05, *p* < 0.05). Teacher’s education and previous experience of teaching an HIV risk reduction program were not associated with the implementation of FOYC.

**Table 2:** Association between teacher’s personal characteristics, training experience and number of activities and sessions taught in the classroom among 110 grade six school teachers

Variables	%	Number of core activities completed (0–30)	Number of all activities completed (0–46)^†^	Number of sessions taught (0–8)
Education level				
Associate degree/teaching certificate	11.3	15.42(9.12)	23.58(14.62)	4.15(2.50)
Bachelor degree	71.7	17.56(8.63)	26.87(13.26)	4.70(2.29)
Master degree	17.0	14.28(7.10)	22.11(10.93)	3.89(1.88)
F test		0.96	0.86	0.83
Total years as teacher and guidance counselor				
1 ~ 10 years	37.6	19.05(9.26)	29.13(14.31)	5.08(2.47)
>10 years	62.4	15.25(7.81)	23.40(12.08)	4.11(2.10)
Student’s *t* test		2.04^*^	1.98^*^	1.95^*^
Attended a FOYC training workshop				
Yes	66.7	16.49(7.41)	25.31(11.42)	4.41(1.98)
No	33.3	17.00(10.61)	25.94(16.42)	4.59(2.84)
Student’s *t* test		0.24	0.18	0.30
Number of days of training attended				
4–5 days	45.8	18.53(7.05)	28.53(11.06)	4.93(1.87)
3 days or less	54.2	14.67(7.34)	22.44(11.11)	3.94(1.98)
Student’s *t* test		2.01^*^	2.05^*^	1.92
Teachers training experience with FOYC				
Completed FOYC training in 2011 and had prior training experience	26.6	19.77(6.93)	30.64(11.13)	5.32(1.87)
Did not complete FOYC training in 2011 and had prior training experience	43.7	14.61(7.72)	22.20(11.75)	3.89(2.06)
No FOYC training prior or in 2011	29.7	16.68(10.45)	25.14(16.29)	4.50(2.79)
F test		2.16	2.37	2.35
Prior experience of teaching HIV risk reduction intervention				
Yes	43.1	15.66(8.57)	24.25(13.17)	4.18(2.28)
No	56.9	17.42(8.51)	26.50(13.23)	4.70(2.28)
Student’s *t* test		0.95	0.78	1.05

Note: Test statistics (*t* and *F* values) were adjusted using the variance inflation factors (VIFs); †include core and review lessons. Numbers outside the parentheses are means (of number of core or all activities completed and number of sessions taught), and numbers in parentheses are standard deviations. *p < 0.05

Teachers who did not perceive that the FOYC intervention was very important for grade six students *in their schools* taught fewer core and all activities and fewer sessions than teachers who perceived that FOYC was very important years (core activities 10.9 vs. 17.5, *t* = 2.46, *p* < 0.05; all activities 16.1 vs. 26.9, *t* = 2.60, *p* < 0.01; and session 2.9 vs. 4.7, *t* = 2.45, *p* < 0.05). By contrast, teachers’ perceptions regarding the importance of HIV prevention for youth or grade six youth *in general* were not associated with the implementation of FOYC. Teachers’ sense of ownership of the FOYC curriculum (e.g., as a “Bahamian intervention”) was not associated with implementation (Table 3).

**Table 3:** Teachers’ perceptions of importance and relevance of FOYC and number of activities and sessions taught among 110 grade six school teachers

Variables	%	Number of core activities completed (0–30)	Number of all activities completed (0–46)^†^	Number of sessions taught (0–8)
Importance of HIV prevention programs for youth in general				
Very important	95.4	16.64(8.56)	25.54(13.19)	4.47(2.29)
Somewhat important	4.6	17.40(8.96)	25.80(14.74)	4.66(2.40)
Student’s *t* test		0.17	0.03	0.16
Importance of HIV prevention programs for grade six youth in general				
Very important	86.9	16.98(8.19)	26.00(12.67)	4.56(2.19)
Somewhat important	13.1	15.92(10.99)	24.08(17.13)	4.23(2.92)
Student’s *t* test		0.37	0.44	0.44
Importance of HIV prevention for Grade 6 youth in your community				
Very important	89.0	17.05(8.43)	26.15(12.99)	4.58(2.26)
Somewhat important	11.0	13.45(9.19)	20.45(14.43)	3.61(2.44)
Student’s *t* test		1.21	1.24	1.22
Importance of FOY for the grade six students in your school				
Very important	86.2	17.51(8.08)	26.90(12.45)	4.70(2.16)
Somewhat important	13.8	10.85(9.73)	16.08(14.81)	2.92(2.61)
Student’s *t* test		2.46^*^	2.60^**^	2.45^*^
FOY curriculum is a Bahamian curriculum				
Very much so	56.9	16.77(7.69)	25.68(11.83)	4.48(2.06)
Somewhat	43.1	16.27(9.86)	24.98(15.16)	4.37(2.62)
Student’s *t* test		0.25	0.24	0.21

Note: Test statistics (*t* values) were adjusted using the variance inflation factors (VIFs); †include core and review lessons. Numbers outside the parentheses are means (of number of core or all activities completed and number of sessions taught), and numbers in parentheses are standard deviations. *p < 0.05; **p < 0.01

### Association between Teacher’s Fidelity of Implementation and Student Outcomes

At baseline, 2,811 students completed program evaluation assessments; 2,742 students completed end of the year assessments. Overall, students’ HIV/AIDS knowledge, reproductive health skills, self-efficacy, and intention to use protection were significantly higher at the end of the year (after being taught some or all of the curriculum) than at the beginning of the school year (knowledge 10.1 vs. 8.4, *t* = 14.14, *p* < 0.001; skills 3.8 vs. 3.5, *t* = 5.39, *p* < 0.001; self-efficacy 1.3 vs. 0.8, *t* = 9.91, *p* < 0.001; and intention 3.2 vs. 2.3, *t* = 9.69, p < 0.001). However, students whose teachers taught fewer than five core activities (equivalent to one complete session) had no improvement in knowledge; students whose teachers taught less than nine core activities (equivalent to two sessions) had no improvement in reproductive health skills, self-efficacy, or intention to use protection.

The results of the mixed-effects models indicate that implementation fidelity was significantly associated with increased scores in the four outcome measures (i.e., knowledge, skills, self-efficacy, and intention). Advanced age was associated with improvement in condom use self-efficacy. Male gender was associated with increased reproductive health skills, self-efficacy, and intention to use condom protection. Higher levels of self-efficacy at baseline were associated with lack of improvement in self-efficacy during the intervention period. Classroom random effects were significant in all four models, indicating a significant variation among classrooms with regard to students’ knowledge of HIV/AIDS, reproductive health skills, self-efficacy, and intention to use condom protection. School random effects were significant for HIV/AIDS knowledge and self-efficacy (Table 4).

**Table 4:** Mixed-effects models assessing the impact of implementation fidelity on students’ outcomes

Variables	Estimated models											
	HIV/AIDS knowledge			Preventive reproductive health skills			Self-efficacy			Intention to use protection		
	β	SE	*t*	β	SE	*t*	β	SE	*t*	β	SE	*t*
*Fixed effect*												
Intercept	8.739	0.796	10.97^***^	3.446	0.412	8.36^***^	1.414	0.388	3.64^***^	2.406	0.680	3.54^***^
Age	0.037	0.064	0.57	0.001	0.036	0.03	0.190	0.032	5.98^***^	−0.060	0.052	−1.16
Gender												
Male	−0.094	0.088	−1.08	0.123	0.049	2.53^*^	0.521	0.043	12.04^***^	0.478	0.070	6.80^***^
Female (ref)												
Baseline student outcome	0.003	0.018	0.16	0.003	0.019	0.15	−0.041	0.021	−1.95^*^	−0.001	0.020	−0.05
Implementation fidelity (number of core activity completed)	0.057	0.018	3.23^**^	0.017	0.006	2.93^**^	0.149	0.073	2.03^*^	0.660	0.208	3.18^**^
*Random effect*												
School^†^	0.395	0.222	1.78^*^	0.007	0.026	0.28	0.090	0.037	2.44^**^	0.066	0.052	1.27
Class (nested within school)^†^	1.155	0.220	5.26^***^	0.171	0.040	4.29^***^	0.061	0.019	3.24^***^	0.277	0.065	4.25^***^

*p < 0.05; **p < 0.01; ***p < 0.001. ^†^z test

### Bivariate Correlation among Factors Influencing Implementation and Student Outcomes

The primary and two secondary indicators of fidelity of implementation were highly correlated with each other (r = 0.98 ~ 0.99, *p* < 0.001). Likewise, HIV/AIDS knowledge, reproductive health skills, self-efficacy, and intention to use protection were significantly correlated (r = 0.22 ~ 0.52, *p* < 0.05). Three indicators of fidelity of implementation were positively associated with student outcomes (e.g., knowledge, skills, and self-efficacy). Teacher’s level of comfort with the FOYC curriculum and perception of the importance of the FOYC curriculum were positively associated with fidelity of implementation (r = 0.27 ~ 0.49, *p* < 0.01). Modifying the FOYC activities and more years as a teacher or guidance counselor were negatively correlated with fidelity of implementation (r = −0.21 ~ −0.38, *p* < 0.05). In addition, teacher’s perception of students’ engagement in the lessons, perception of importance of FOYC, and attendance at the training workshop were negatively associated with modifying FOCY activities (r = −0.23 ~ −0.35, *p* < 0.05) (Table 5).

**Table 5:** Correlation coefficients of indicator variables of teacher’s fidelity of implementation, factors influencing fidelity of implementation, and students’ outcome variables

Variables	1	2	3	4	5	6	7	8	9	10	11	12	13	Mean	SD
Fidelity of implementation															
1. Number of core activities completed (0–30)	1.00													16.34	8.75
2. Number of all activities completed (0–46)	0.99^***^	1.00												24.87	13.60
3. Number of sessions taught (0–8)	0.99^***^	0.98^***^	1.00											4.38	2.34
Factors influencing fidelity of implementation															
4. Perception of importance of FOYC	0.27^**^	0.27^**^	0.28^**^	1.00										1.86	0.35
5. Modifying FOYC activities/lessons	−0.36^***^	−0.36^***^	−0.38^***^	−0.23^*^	1.00									0.22	0.24
6. Comfortableness with FOYC lessons	0.45^c^	0.44^c^	0.49^c^	0.20^a^	−0.53^c^	1.00								2.64	0.41
7. Years as teacher or guidance counselor	−0.21^*^	−0.23^*^	−0.22^*^	−0.13	−0.02	0.01	1.00							1.93	0.84
8. Students’ engagement in FOYC lessons	0.16	0.17	0.17	0.06	−0.35^***^	0.34^***^	0.24^*^	1.00						2.83	0.24
9. Attendance of FOYC training workshop	0.08	0.09	0.10	0.12	−0.27^**^	0.12	0.13	0.18^*^	1.00					1.61	1.57
Students’ outcomes															
10. HIV/AIDS Knowledge	0.44^***^	0.43^***^	0.43^***^	0.24^*^	−0.13	0.17	−0.04	0.04	−0.04	1.00				10.02	1.30
11. Preventive reproductive health skills	0.38^***^	0.38^***^	0.39^***^	0.22^*^	−0.03	0.08	0.06	0.13	0.13	0.52^***^	1.00			3.78	0.43
12. Self-efficacy	0.19^*^	0.17^*^	0.17^*^	0.21^*^	−0.13	0.15	−0.01	−0.03	0.07	0.26^**^	0.35^***^	1.00		1.30	0.43
13. Intention to use condom	0.10	0.10	0.09	0.35^***^	−0.23^*^	0.34^***^	−0.05	0.15	0.16	0.39^***^	0.30^***^	0.22^*^	1.00	3.12	0.60

Note: ^*^ *p* < 0.05; ^**^ *p* < 0.01; ^***^ *p* < 0.001. SD = Standard deviation

### Structural Equation Modeling

**Fig. 2:**
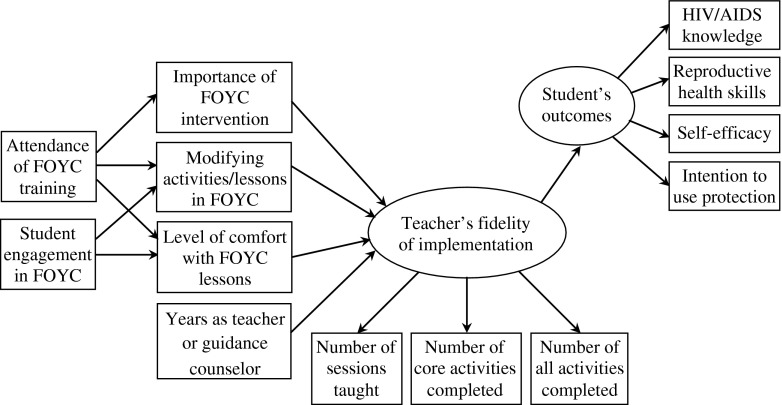
Hypothesized initial model of the relationships among factors influencing teacher’s fidelity of implementation of FOYC intervention and student’s knowledge, skill, perception, and intention outcomes

Initially, a hypothetical model was developed based on a synthesis of the empirical literature (Fig. 2). The model posits that teacher’s level of comfort with FOYC lessons, teaching experience (fewer years as a teacher), and perception of importance of FOYC have a direct positive effect, while teacher’s modification of FOYC lessons has a negative effect on teacher’s fidelity of implementation; implementation fidelity in turn affects student’s outcomes. Teacher’s level of comfort and teacher’s modification of FOYC lessons are influenced by whether they received training on FOYC curriculum and student engagement in FOYC.

**Fig. 3:**
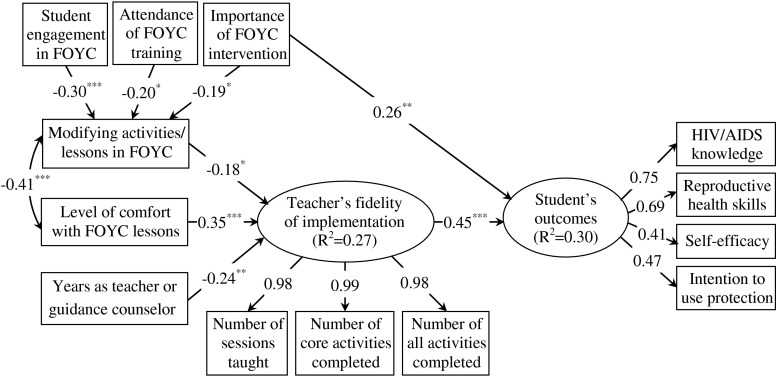
Revised structural model of the relationships among factors influencing teacher’s fidelity of implementation of FOYC intervention and student’s knowledge, skill, perception, and intention outcomes. (Model fit: CFI = 0.942; TLI = 0.924; RMEA = 0.08; SRMR = 0.06; Chi-square/DF = 2.21)

The revised structural model demonstrated the relationship among factors and their direct and indirect effects on fidelity of implementation and student outcomes (Fig. 3). There were six manifest exogenous variables and two latent endogenous variables (e.g., fidelity of implementation and student’s outcomes) in the model. The overall fit of the revised model was good (CFI = 0.94; TLI = 0.92; RMEA = 0.08; SRMR = 0.06; *χ* ^2^/df = 2.21). The analysis revealed an R^2^ value of 0.27 for fidelity of implementation and of 0.30 for student’s outcomes.

In the revised model, comfort with the FOYC curriculum predicted high-level fidelity of implementation which in turn predicted better student outcomes (HIV/AIDS knowledge and/or reproductive health skills and/or self-efficacy). More years as a teacher or guidance counselor were negatively associated with fidelity of implementation, which was in turn associated with poorer student outcomes. Teachers who did not perceive that the FOYC intervention was important for grade six students, who had attended only a portion of the FOYC teacher training workshop or who perceived that their students were not engaged in the FOYC lessons tended to modify FOYC activities/lessons, which in turn exerted a negative influence on fidelity of implementation defined as number of core activities, as well as all activities and sessions taught. In addition, teacher’s perception of the importance of the FOYC intervention and fidelity of implementation had a direct positive effect on student outcomes. The Sobel test of mediation effect indicated that fidelity of implementation mediated the relationship between teacher’s comfortableness with FOYC lessons, modifying the FOYC activities/lessons, years as teacher or guidance counselor, and student’s outcome (z = 3.01, *p* < 0.01; z = 196, *p* < 0.05; z = 2.54, *p* = 0.01).

## Discussion

Based on the data from a large-scale national implementation study, the present study identified the factors that influence teachers to implement the FOYC intervention with fidelity in school settings and examined the interrelationships among these influencing factors and how these processes ultimately influence program outcomes. The findings reveal that teacher’s level of comfort with FOYC curriculum is the strongest predictor of fidelity of implementation. Teacher’s perceptions that the FOYC intervention is important for their students and that the students were engaged in the FOYC lesson, as well as teacher attendance at a FOYC teacher training workshop protected against modifying the FOYC activities, which in turn exerts a positive influence on fidelity of implementation. Finally, teacher’s fidelity of implementation is associated with improved student outcomes.

Our findings that teacher’s perception of the importance of the FOYC intervention and level of comfort with FOYC curriculum were positively related to fidelity of implementation are consistent with prior studies (Beets et al. 2008; Rohrbach et al. 1993). Teacher’s perception of the importance of HIV prevention and self-confidence in teaching FOYC are potential modifiable factors related to program delivery (Dusenbury et al. 2003). Therefore, efforts should be directed towards enhancing teachers’ competency regarding teaching the intervention curriculum and shaping teachers’ attitudes towards and belief about the importance of HIV prevention programs in their communities through pre-implementation teacher training.

Data in the present study indicate that teachers who completed a FOYC training workshop implemented a greater proportion of the FOYC curriculum, including the core elements. However, teachers who only attended part of a training workshop did not perform better than teachers who did not attend a training workshop. This finding is consistent with prior research suggesting that only intensive training was positively associated with higher fidelity of implementation (Perry et al. 1990). Thus, efforts should be made to ensure a full teacher participation in the training workshops. Alternatively, the finding may reflect that these teachers were ambivalent about teaching FOYC, especially as they came to understand the nature of the curriculum after the first day or two of the workshop and therefore stopped attending. This interpretation underscores the importance of addressing teacher concerns forthrightly and early in workshops.

Our study shows that modifying the FOYC activities was negatively associated with fidelity of implementation and student outcomes. Such a finding is inconsistent with some studies that have found that some modification by the teachers was associated with improved student outcomes (McGrew et al. 1994). In our study, teachers who reported having modified the FOYC activities actually changed core activities from the outlined procedures, which can have a negative impact on program effectiveness. In addition, teachers’ sense of “ownership” of the curriculum (i.e., perceiving FOYC as a “Bahamian intervention”) was not associated with implementation fidelity, which is inconsistent with the previous studies indicating a positive association (Draper et al. 2010; Dusenbury et al. 2003). Future research is needed to study how the teachers changed the FOYC activities and reexamine the association of fidelity of implementation with modifying the FOYC activities and teacher’s sense of “ownership” of the FOYC curriculum.

Teachers in general only taught half of the FOYC curriculum in this phase of national implementation. In the original randomized, controlled trial of the FOYC intervention, almost all the teachers taught ten sessions of the FOYC curriculum. During the FOYC trial, for the few teachers who expressed discomfort in teaching one or two sessions (e.g., condom use demonstration), assistance was provided by the FOYC workshop trainers. The extant literature suggests that when teachers are aware that they have backup resources, they are more likely to continue in their efforts (Dusenbury et al. 2003). It is possible that the FOYC workshops for national implementation did not adequately articulate this option. Therefore, if a school system is willing to offer support in a non-judgmental fashion, this option should be articulated during the training workshop. Such articulation of support might also reinforce the teacher’s perception of the support of the educational system for the curriculum.

Although the MOE fully supported the implementation of the FOYC intervention among grade six students, teacher’s perception of school-level support (school administrators) varied from school to school. For example, some teachers said the FOYC curriculum was not a top priority in their schools as evidenced by scheduling it for Friday afternoons which are often utilized for outside presentations and non-academic activities, and FOYC lessons were frequently canceled (Knowles et al. 2012).

There are several potential limitations in this study. First, the measure of fidelity of implementation relied on teachers’ self-report and thus may not have been accurate. However, trained observer’s independently observed and assessed approximately 20 % of each teacher’s classes. The teacher and observer reports on activities covered these sessions were compared to determine the level of agreement; in general, we found that the observer-teacher agreement was high (86 %), lending credence to the teacher self-reports. Second, organizational characteristics such as support by the principal, school administrators perception of importance of HIV intervention, and school’s readiness to implement the intervention were not collected as part of the first wave of national implementation (These measures will be collected in the second wave of national implementation). Third, some of the questions regarding teacher’s perception were too general (e.g., “in general how important do you think prevention programs are for youth?”). Finally, our study only captured three components of implementation fidelity: adherence to the core activities; completeness of delivery (number of all activities and sessions taught); and some aspects of participant responsiveness (e.g., teacher’s perception of student engagement and student changes in knowledge, perceptions, skills, etc.). We recognize that there are at least two other components (quality of program delivery and program differentiation) (Dusenbury et al. 2003). Future studies are needed to measure all five dimensions of fidelity in order to provide a complete picture of program implementation.

## Conclusions

This study provides an integrated understanding of the relationships between teacher characteristics, training experience and perceptions with fidelity of implementation, and student outcomes. These findings have significant implications for future implementation efforts. First, pre-implementation teacher training should encourage full attendance by the teacher at the workshops to increase the teacher’s comfort level with and/or competence in teaching the intervention curriculum. For those teachers who fail to attend some or all of the training workshop, other mechanisms might be considered to provide a program support (e.g., teachers who attended the training could be asked to review some intervention lessons with those who did not, videos of the training could be made and shared), potentially improving program implementation. Second, the pre-implementation assessment of teacher attitudes towards and perceptions about HIV prevention programs can help to identify “high-risk” teachers who may be less likely to deliver the program with fidelity. Special attention should be paid to this subgroup of program providers including providing additional evidence of program effectiveness. Finally, clear indication of support by the local school administrators for the curriculum is important. Although considerable effort is required to provide an adequate support for teachers to teach intervention programs as intended, an evidence-based HIV prevention intervention can be successfully implemented in schools at a national level after proper preparation of implementation.
